# Efficient Inverted Organic Solar Cells Based on a Fullerene Derivative-Modified Transparent Cathode

**DOI:** 10.3390/ma10091064

**Published:** 2017-09-11

**Authors:** Yifan Wang, Hailin Cong, Bing Yu, Zhiguo Zhang, Xiaowei Zhan

**Affiliations:** 1 Institute of Biomedical Materials and Engineering, College of Chemistry and Chemical Engineering, Qingdao University, Qingdao 266071, China; wangyifan@qdu.edu.cn (Y.W.); yubingqdu@yahoo.com (B.Y.); 2 Laboratory for New Fiber Materials and Modern Textile, Growing Base for State Key Laboratory, College of Materials Science and Engineering, Qingdao University, Qingdao 266071, China; 3 Beijing National Laboratory for Molecular Sciences and Key Laboratory of Organic Solids, Institute of Chemistry, Chinese Academy of Sciences, Beijing 100190, China; zgzhangwhu@iccas.ac.cn; 4 Department of Materials Science and Engineering, College of Engineering, Peking University, Beijing 100871, China; xwzhan@pku.edu.cn

**Keywords:** organic solar cell (OSC), cathode buffer layer (CBL), transparent conducting material, fullerene derivative, ZnO

## Abstract

Indium tin oxide (ITO) is a transparent conductive material which is extensively used in organic solar cells (OSCs) as electrodes. In inverted OSCs, ITO is usually employed as a cathode, which should be modified by cathode buffer layers (CBLs) to achieve better contact with the active layers. In this paper, an amine group functionalized fullerene derivative (DMAPA-C_60_) is used as a CBL to modify the transparent cathode ITO in inverted OSCs based on PTB7 as a donor and PC_71_BM as an acceptor. Compared with traditional ZnO CBL, DMAPA-C_60_ exhibited comparable transmittance. OSCs based on DMAPA-C_60_ show much better device performance compared with their ZnO counterparts (power conversion efficiencies (PCEs) improved from 6.24 to 7.43%). This is mainly because a better contact between the DMAPA-C_60_ modified ITO and the active layer is formed, which leads to better electron transport and collection. Nanoscale morphologies also demonstrate that the surface of DMAPA-C_60_-modified ITO is plainer than the ZnO counterparts, which also leads to the better device performance.

## 1. Introduction

Bulk-heterojunction (BHJ) organic solar cells (OSCs) have been regarded as one of the most advanced photovoltaic technologies because of their advantages over traditional silicon solar cells, which include low cost, light weight, and flexibility [1,2,3,4]. Nowadays, OSCs based on fullerene as acceptors or non-fullerene organic materials as acceptors have achieved power conversion efficiencies (PCEs) of over 11% [5,6,7,8,9]. Inverted OSCs, which are mostly based on conductive indium tin oxides (ITO) as cathodes and silver (Ag) as anodes, possess major advantages of enhanced device performance as well as increased stability compared with conventional OSCs [10,11]. In recent years, inverted OSCs have developed rapidly, mostly benefiting from the development of novel donor and acceptor materials and interfacial engineering [12]. Electrode-semiconducting active layer contacts in organic electronics are plagued by interfacial barriers for charge transfer due to poor alignment between the electrode work function and the Fermi level of the semiconductor [13]. As a result, the cathode buffer layer (CBL) between the active layer and conductive ITO cathode plays an important role in the performance of inverted OSCs because it can decorate the ITO cathode and promote the electron transfer and extraction process [14,15].

**Figure 1:**
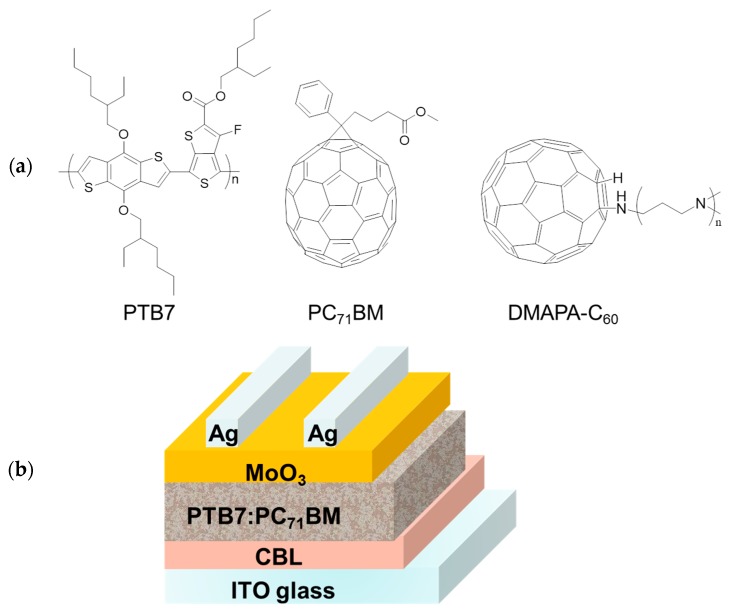
(**a**) Chemical structures of PTB7, PC_71_BM, and DMAPA-C_60_; (**b**) Device structure of the inverted OSC.

CBLs can be grouped into two categories: inorganic and organic materials. Inorganic materials are commonly transition-metal oxides, such as CsCO_3_ [16,17], TiO_x_ [18,19], and ZnO [20,21,22]. Different from inorganic CBLs, organic CBLs exhibit the advantage of structural tunability, which could offer more opportunities of realizing better interface modification and high-performance fully solution-processed OSCs [23,24]. In view of this, various types of organic CBLs have drawn much attention and have been recently developed. For example, water (alcohol)-soluble conjugated polymers such as poly[(9,9-bis(3′-(*N*,*N*-dimethylamino)propyl)-2,7-fluorene)-alt-2,7-(9,9-dioctylfluorene)] (PFN) and its derivatives [25,26] have been successfully utilized as CBLs in inverted OSCs and have been proved to be comparable to or even better than the conventional ZnO CBL. Other organic CBLs such as polyethylenimine ethoxylated (PEIE) [27] and titanium (diisopropoxide) bis(2,4-pentanedionate) (TIPD) [28] have also been proved to be potential CBLs in inverted OSCs with performances better than ZnO CBLs. Recently, fullerene-based materials have been designed as a new type of CBL. Fullerene-based materials are considered to be ideal candidates for CBLs, because their structures are similar to the conventionally used fullerene acceptors, which could smoothly bridge electrons transporting from the fullerene acceptor to the cathode [29,30,31,32]. For example, Alex and co-workers reported that, with a new kind of fullerene derivative Bis-OMe fulleropyrrolidinium iodide (FPI) blended PEIE CBL in inverted OSCs, a much better performance is achieved compared with using only PEIE as the CBL [33]. An amine group functionalized fullerene complex (DMAPA-C_60_, Figure 1a) was successfully synthesized as an ideal candidate for CBLs to modify the Al cathode in conventional OSCs [34]. Its terminal amino group could potentially provide a dipole moment, just as the amino group does in PFN. Furthermore, the lowest unoccupied molecular orbital (LUMO) energy level is estimated to be 3.58 eV, close to that of PC_71_BM (about 3.85 eV). The highest occupied molecular orbital (HOMO) energy level is estimated to be 5.52 eV, which is low enough to efficiently block holes from various donors.

In this paper, we demonstrate that DMAPA-C_60_ can be independently used as a CBL to modify the transparent conductive cathode ITO in inverted OSCs based on poly{4,8-bis[(2-ethylhexyl)oxy]benzo[1,2-*b*:4,5-*b*′]dithiophene-2,6-diyl-alt-3-fluoro-2-[(2-ethylhexyl)carbonyl]thieno[3,4,[3,4-*b*]thiophene-4,6-diyl} (PTB7) as a donor and PC_71_BM as an acceptor. Compared with the traditional ZnO CBL, a much better device performance was realized (PCE improved from 6.24 to 7.43%).

## 2. Results and Discussion

### 2.1. Absorption and Transmittance

**Figure 2:**
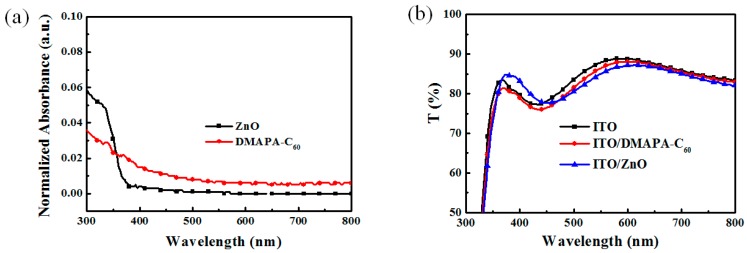
(**a**) UV-Vis absorption spectra of ZnO and DMAPA-C_60_ films; (**b**) Transmittance spectra of ITO, ITO/DMAPA-C_60_, and ITO/ZnO films.

To modify ITO cathodes in inverted OPV devices, the buffer layers should be as transparent as possible, which can guarantee the more sunlight will penetrate the cathode and be absorbed by the active layers. The normalized spectra of optical absorption of the ZnO buffer layer and DMAPA-C_60_ buffer layer are shown in Figure 2a. Both ZnO and DMAPA-C_60_ buffer layers exhibit very weak absorption at 400~800 nm. The transmittance spectra of the ITO layer, ZnO-modified ITO layer, and DMAPA-C_60_-modified ITO layer are shown in Figure 1b. They all show excellent transmittance of over 85%, demonstrating the excellent light transmissivity of the buffer layers, which is suitable for modifying the ITO cathode.

### 2.2. Photovoltaic Performances

**Figure 3:**
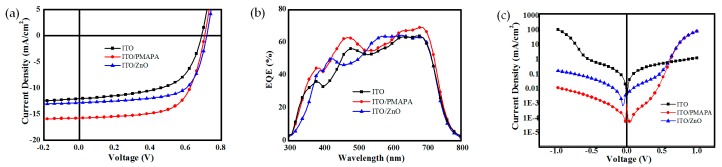
(**a**) *J*-*V* curves, (**b**) EQE curves and, (**c**) Dark *J-V* curves of OSCs based on different CBLs.

We fabricated BHJ OSCs with a structure of ITO/CBL/PTB7:PC_71_BM/MoO_3_/Ag using classical low-bandgap polymer PTB7 as a donor material and PC_71_BM as an acceptor material. Table 1 shows the device parameters of DMAPA-C_60_-based OSCs with different DMAPA-C_60_ concentrations, indicating the thickness dependence of these devices. After optimization, OSCs with 2 mg/mL DMAPA-C_60_ shows the best performance with a PCE of 7.43%. Figure 3a shows the current density-voltage (*J-V*) curves of the OSCs based on different CBLs (under the illumination of AM1.5G, 100 mW·cm^−2^). The corresponding device parameters are summarized in Table 2. The devices with bare ITO as a cathode shows the lowest PCE of 4.93%, with a very small *J*_SC_ of 12.0 mA·cm^−2^ and an fill factor (FF) of only 58.5%. This can be ascribed to the direct contact of ITO with the active layer, leading to a large series resistance (*R*_S_) and a small parallel resistance (*R*_SH_). When we utilize ZnO CBL to modify the ITO cathode, the device performance enhances significantly, with *V*_OC_ improving from 0.69 V to 0.71 V, *J*_SC_ from 12 mA·cm^−2^ to 12.8 mA·cm^−2^, FF from 58.5 to 66.9%, and PCE from 4.93 to 6.24%. Simultaneously, it can be seen that with ZnO as the CBL, the *R*_S_ decreases from 7.03 Ω·cm^−2^ to 6.22 Ω·cm^−2^, indicating that better contact is formed between the cathode and the active layer, and carrier trapping and recombination inside the device is reduced. With DMAPA-C_60_ as the CBL, an even higher PCE of 7.43% is achieved, mostly due to the enhancement of *J*_SC_ from 12.8 mA·cm^−2^ to 15.7 mA·cm^−2^. This is consistent with the fact that the *R*_S_ value (3.50 Ω·cm^−2^) of the DMAPA-C_60_ device is much smaller than that of the ZnO device (6.22 Ω·cm^−2^). The external quantum efficiency (EQE) curve is shown in Figure 2b. The *J*sc values integrated from the EQE spectra are 11.9, 15.1, 12.3 mA·cm^−2^ for the no-CBL, DMAPA-C_60_-based, and ZnO-based devices, respectively, which is consistent with the values shown in Table 2.

Figure 3c shows the dark *J*-*V* curves of these three kinds of devices. It can be seen that under the reverse voltage, the device with the DMAPA-C_60_-modified ITO cathode shows the lowest dark current, which suggests after adding the DMAPA-C_60_ CBL, the bimolecular recombination is reduced remarkably, leading to the enhancement of the light current. In addition, *V*oc can be calculated based on the well-known equation:

(1) $$V_{\mathrm{OC}}\;\approx \;\frac{nkT}{q}\ln\left(\frac{J_{\mathrm{SC}}}{J_{0}}\right),$$

where *q* is the elemental charge, *k* is the Boltzmann coefficient, *T* is the temperature, and *J*_0_ is the reverse saturation current. According to Equation (1), the smaller the *J*_0_, the larger the *V*_OC_. As a result, the *V*_OC_ of the DMAPA-C_60_ device slightly increases compared with the other two devices. So, from the above, we can draw a conclusion that DMAPA-C_60_ is a promising CBL to modify ITO cathodes in inverted OSCs.

### 2.3. Morphology

**Figure 4:**
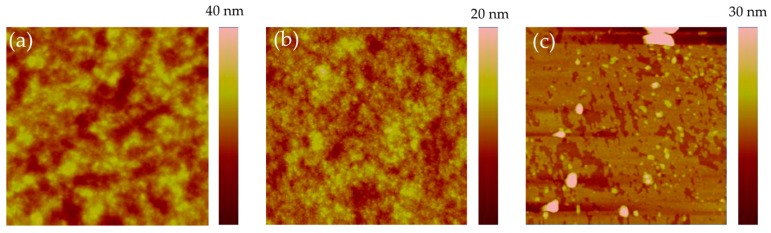
AFM height images (3 × 3 μm^2^) of (**a**) ITO, (**b**) DMAPA-C_60_-decorated ITO, and (**c**) ZnO-decorated ITO.

Atomic force microscopy (AFM) was employed to investigate the nanoscale morphology of ITO films modified by different CBLs. The height images are shown in Figure 4. It can be observed that the bare ITO film exhibits a relatively rough surface with the root mean square (RMS) roughness of 10.10 nm. After decorating with ZnO, the RMS decreases to 7.25 nm. With the modification of DMAPA-C_60_, a much smoother surface is obtained with an RMS of only 4.57 nm. Thus, devices with DMAPA-C_60_ as CBLs possess better contact between the active layers and cathodes, suppressing charge recombination in the interface. This could be another reason to explain the improvement of PCE of the DMAPA-C_60_ device.

### 2.4. Stability

**Figure 5:**
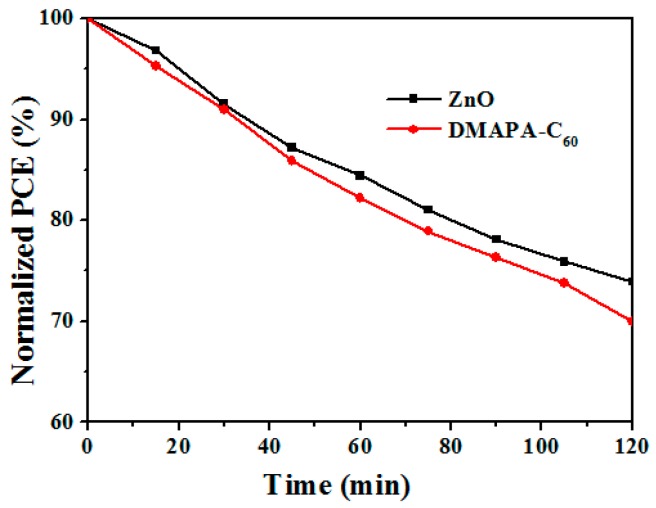
Stability curves of ZnO and DMAPA- C_60_ devices under continuous heating at 130 °C for 120 min.

After heating at 130 °C for 120 min, the PCE of ZnO-based devices decays from 6.24 to 4.61%, preserving 73.9% of its original value. Replacing ZnO with DMAPA-C_60_, the PCE decays from 7.43 to 5.2%, showing 70% of its original value, which indicates that DMAPA-C_60_-based devices exhibit similar good thermal stabilities as ZnO-based devices (as shown in Figure 5).

## 3. Materials and Methods

### 3.1. Materials

Unless stated otherwise, solvents and chemicals were obtained commercially and were used without further purification. DMAPA-C_60_ was synthesized according to Reference [34].

### 3.2. Preparation of ZnO

Zinc acetate dihydrate [Zn(CH_3_COO)_2_·2H_2_O] was first dissolved in anhydrous 2-methothyethanol (0.5 M concentration) and then ethanolamine was added to the solution as sol stabilizer (0.5 M concentration) followed by thorough a mixing process with a magnetic stirrer for 12 h.

### 3.3. Fabrication of the OSCs

Organic solar cells were fabricated with the following structures: ITO/PTB7:PC_71_BM/MoO_3_/Ag, ITO/ZnO/PTB7:PC_71_BM/MoO_3_/Ag, ITO/DMAPA-C_60_/PTB7:PC_71_BM/MoO_3_/Ag. Patterned ITO glass (sheet resistance = 15 Ω·□^−1^) was precleaned in an ultrasonic bath with deionized water, acetone, and isopropanol (each for 15 min). Then different CBLs were spin-coated on ITO. For ZnO as the CBL, the prepared ZnO sol-gel was spin-coated on the ITO substrate with 3000 rpm and then annealed at 200 °C for 1 h in the air. For DMAPA-C_60_ as the CBL, methanol solution of DMAPA-C_60_ was deposited atop the ITO substrate at 3000 rpm for 30 s. Then the active layer was formed by spin-coating from o-dichlorobenzene (o-DCB) solution containing 10 mg·mL^−1^ PTB7 and 15 mg·mL^−1^ PC_71_BM at 1000 rpm for 1 min. 1,8-Diiodooctane with a 3% volume ratio was added to the o-DCB solutions and stirred before use. A MoO_3_ (ca. 5 nm) and silver layer (ca. 80 nm) was then evaporated onto the surface of the active layer under vacuum (ca. 10^−5^ Pa) to form the positive electrode. The active area of the device was 4 mm^2^.

### 3.4. Characterizations

Current density-voltage characteristics were measured inside a N_2_-filled glove box by using a source meter (2400, Keithley Instruments, Cleveland, OH, USA) controlled by a LabVIEW program (National Instruments, Austin, TX, USA) in the dark and under white light illumination (100 mW·cm^−2^). The light intensity was calibrated using a silicon photodiode with a KG5 filter (S1133, Hamamatsu Photonics, Hamamatsu, Japan). The EQE spectra were recorded using a Solar Cell Spectral Response Measurement System QE-R3011 (Enlitech Co., Ltd., Kaohsiung, Taiwan). The light intensity at each wavelength was calibrated by using a standard single crystal Si photovoltaic cell. Thin-film UV-vis absorption and transmission curves were recorded on a JASCO V-570 spectrophotometer. The nanoscale morphology of the blended films was observed using a Veeco Nanoscope V atomic force microscope in the tapping mode. The stability test of solar cells was carried out under continuous heating at 130 °C and the *J*-*V* was measured using a B2912A source meter (Angilent Technologies, Palo Alto, CA, USA) every 15 min for a total of 120 min under AM 1.5 G illumination. These tests were carried out in the glove box.

## 4. Conclusions

In conclusion, an easy-accessible fullerene derivative, DMAPA-C_60_, was developed as a CBL to modify the transparent conducting cathode ITO in inverted OSCs based on a PTB7:PC_71_BM system. Compared with traditional ZnO CBLs, DMAPA-C_60_ exhibited comparable transmittance. Meanwhile, OSCs based on DMAPA-C_60_ showed much better device performance compared with their ZnO counterparts (PCE improved from 6.24 to 7.43%). This is mainly caused by the much lower Rs, that is to say, a better contact between the DMAPA-C_60_-modified ITO and the active layer is formed and a better electron collection at the cathode is realized. Nanoscale morphologies also demonstrated the plainer surface of DMAPA-C_60_-modified ITO compared with the ZnO counterparts, which leads to the lower Rs and higher PCE. The success of DMAPA-C_60_ as a CBL layer indicates that the amine groups functionalized fullerene derivatives could be very promising in replacing conventional ZnO CBLs for low-cost, high-performance inverted OSCs.

## Figures and Tables

**Table 1:** Device performances of OSCs based on DMAPA-C_60_ with different DMAPA-C_60_ concentrations.

DMAPA-C_60_ (mg/mL)	*V*_OC_ (V)	*J*_SC_ (mA·cm^−2^)	FF (%)	PCE (%)	
				Best	Average ^a^
1	0.69	14.2	57.3	5.61	5.48
1.5	0.69	14.3	63.4	6.26	6.1
2	0.70	15.7	65.7	7.43	7.27
2.5	0.70	15.4	61.4	6.62	6.45
3	0.69	14.8	62.9	6.42	6.29

^a^ The average PCE was obtained from 25 devices.

**Table 2:** Device performances of OSCs based on different CBLs.

Structure	*V*_OC_ (V)	*J*_SC_ (mA·cm^−2^)	FF (%)	PCE (%)		R_S_ (Ω·cm^−2^)	R_SH_ (Ω·cm^−2^)
				Best	Average ^a^		
ITO	0.69	12.0	58.5	4.93	4.59	7.03	427.3
ITO/DMAPA-C_60_	0.70	15.7	65.7	7.43	7.27	3.50	580.2
ITO/ZnO	0.71	12.8	66.9	6.24	6.01	6.22	685.4

^a^ The average PCE was obtained from 25 devices.
